# Academic Performance, Health and Support Needs: Comparing Foster Care Alumni and Peers in Higher Education in Norway

**DOI:** 10.3390/ijerph21111470

**Published:** 2024-11-05

**Authors:** Stine Lehmann, Mari Hysing, Børge Sivertsen

**Affiliations:** 1 Department of Clinical Psychology, Faculty of Psychology, University of Bergen, 5020 Bergen, Norway; 2 Department of Psychosocial Science, Faculty of Psychology, University of Bergen, 5020 Bergen, Norway; mari.hysing@uib.no; 3 Department of Health Promotion, Norwegian Institute of Public Health, 5808 Bergen, Norway; borge.sivertsen@fhi.no; 4 Department of Research & Innovation, Helse-Fonna HF, 5525 Haugesund, Norway

**Keywords:** higher education, foster care, student, mental health, somatic health, educational needs

## Abstract

The completion rates of higher education are low among young adults with a history of foster care. Understanding how students from foster care alumni fare is imperative for facilitating attainment and preventing drop-out. The aim of this study was to compare students from foster care alumni and the general student population by examining (1) sociodemographic characteristics, academic functioning and need for special assistance; (2) contextual factors important for student life; and (3) mental and somatic health, loneliness, life satisfaction and use of cannabis and alcohol. Data comprised self-reports from the Norwegian national survey Students’ Health and Well-being Study 2022. Reports from foster care alumni students (*n* = 508) were compared to those of the general student sample (*n* = 59,036). Compared to the general sample, twice as many foster care alumni students reported receiving or needing special assistance in their studies. Moreover, they reported substantially higher economic burden than their student peers, by higher frequency of work income, more financial worries, and less parental financial support. Foster care alumni students also reported a higher load of informal care responsibilities, poorer mental and somatic health, more loneliness and lower life satisfaction. Foster care alumni students fare comparably well in terms of grades and study progression despite the reported economic burden and impaired health and well-being. As these are factors shown to impact study completion, policies need to be put in place to ensure that care-experienced students receive adequate support through their young adulthood and specifically in higher education.

## 1. Introduction

To ‘*ensure inclusive and equitable quality education and promote lifelong learning opportunities for all*’ is one of the United Nations’ sustainability goals (un.org). Improved health, increased earning potential and life satisfaction are among the benefits of achieving higher education at the individual level [1]. The societal benefits include lower unemployment rates, increased tax revenues, greater intergenerational mobility, deeper civic and volunteer participation, and lessened dependency on social services. Students in foster care are defined as one of the targeted minority groups that are at risk of restricted access to and in turn are underrepresented in higher education [2]. In Norway, there are two main forms of out of home placements. Placement in foster families is the preferred option, where children and youth are placed in families who are engaged by and receive follow-up by the child welfare services (CWSs). For a smaller group of adolescents, residential youth care (RYC) is an alternative. RYC institutions are often small units with expertise in, for example, conduct problems, or problems with substance use. In this study, we aim to increase the knowledge of how students in higher education with a history of out-of-home care (hereafter foster care alumni) fare compared to their peers in the general student population.

The rate of foster youth who complete higher education is lower than their peers. In the US, the estimated percentage of foster youth who graduate from college ranges from 1 to 11%, which is in contrast to approximately 30% in the general population [3]. This trend is also found in Scandinavia; while 34% of males and 44% of females in the majority population had obtained a college degree at 26, this was true for only 8 and 13% of the foster care alumni [4]. One factor that could account for lower education levels is the relatively low frequency of entry into higher education. In Norway, six years after completing mandatory education 47.9% of the general population is enrolled in higher education, while this is true for only 11.8% of young adults with a history of out-of-home care [5]. Among young adults with a history of RYC in Norway, only 6% had completed 1 or more years of higher education [6]. This may in part be explained by the lack of qualifications needed for entry. Registry data show that among adults who have received any form of intervention from the child welfare services during their childhood, only 40% completed upper secondary school compared to 81% in the general population [7].

Among foster care alumni that enter higher education, studies indicate that they often need longer time to complete their degree [8]. As a group, foster care alumni students have lower graduation rates than the general population [9,10], which is also true compared to low-income- and first-generation students [11]. For foster care alumni, dropouts from college often take place as early as during the first year of higher education [12,13].

For students with a history of foster care, several factors may delay or hinder their grade completion. Life circumstances preceding enrollment or during the course of higher education may play a role in predicting college completion. In general, parents’ level of education is associated with their offspring’s educational attainment [4,14]. For foster care alumni, especially for females, educational attainment is associated with foster parents’ educational status [15]. Financial hardship and subsequent workload beside studies have been found to predict the non-completion of college for foster care alumni [16]. Among foster care alumni who succeed in their pursue of higher education, actively seeking help in navigating the educational system [17], a sense of autonomy, as well as social and financial support [18] are highlighted as important.

Ongoing mental health problems are common among young people who have experienced multiple potential traumatic events during their childhood [19]. It is well established that children and youth in out-of-home care have a high prevalence of mental disorders [20,21,22], poor physical health [23] and impaired quality of life [24,25]. Mental health is an important predictor for progression and completion of higher education [26]. Indeed, mental health problems have been shown to be a key predictor for disrupted educational pathways for youth in out-of-home-care [27]. Also, the fear of stigma and low self-esteem related to being in foster care may be burdensome [28,29]. However, we have limited knowledge on the mental and somatic health status and well-being among the selected group of young adults who have experienced foster care and who have succeeded in entering higher education.

High risk substance use is associated with functional outcomes, including higher education [30]. As substance use disorders are known to be more common among foster care alumni [31], this may be a relevant risk factor for the completion of higher education in this group. However, we lack systematic knowledge of whether substance use is more prevalent among foster care alumni who are enrolled in higher education. For students, having informal care responsibilities also increases the risk of delayed study progress and failed exams compared to students without such responsibilities [32]. Among foster care alumni, one may expect that the burden of informal care responsibilities is high due to frequent mental health and addiction problems among their biological parents combined with a lack of parental support given that foster parents no longer have a formal role as parents for the young adult. Loneliness is an increasing public health problem among young adults [33], and social connectedness has been previously shown to be a predictor of post-college outcomes [34]. To our knowledge, there is limited research on informal care responsibilities and loneliness among foster care alumni students.

To tailor preventive measures against drop-out among foster care alumni students, we need systematic knowledge of contextual factors known to affect academic attainment as well as of the mental and somatic health needs in this group relative to their student peers. With use of the self-report from The Students’ Health and Well-being (SHoT) Study 2022, our aim is to examine how foster care alumni students fare compared to their peers in the general student population in Norway. We assess (1) sociodemographic characteristics, academic functioning (grade, fail, progression) and special assistance/accommodations; (2) contextual and factors important for student life; responsibilities as young carers, part-time work, economic strain, parental economic support, parents’ educational attainment; and (3) self-reported mental and somatic health, loneliness, life satisfaction and substance use.

## 2. Material and Methods

### 2.1. Procedure and Participants

The SHoT study is a national survey targeting all students in Norwegian higher education. Since its inception in 2010, four surveys have been conducted. This report use data from the latest wave, which was completed in 2022. Detailed information about the SHoT study has been outlined in a previous publication [35]. The SHoT2022 was initiated by the three largest student welfare organizations representing all student welfare organizations in Norway. The study was conducted as a joint effort between these student welfare organizations and the Norwegian Institute of Public Health (NIPH). Data collection for SHoT2022 took place from February to April 2022 and involved full-time students enrolled in higher education across Norway. A total of 169,572 students enrolled at the time were invited to participate with 59,554 completing the web-based questionnaires, resulting in a response rate of 35.1%. All data used in this study are derived from the web-based questionnaires and are described in detail below.

### 2.2. Instruments

#### 2.2.1. Placement Characteristics

We designed a structured questionnaire to explore participants’ early caregiving environments. Initially, participants were asked to identify all applicable caregiving arrangements from a list that included biological parents, foster parents, adoptive parents, and RYC. For those who reported having lived in foster homes or RYC, we gathered further information to assess the stability and variability of their out-of-home placement history. This included determining the total number of different foster homes or RYC institutions they had lived in, their age at first placement, the duration of their longest stay, and the frequency of moves between different foster homes or RYC-institutions.

#### 2.2.2. Sociodemographic Information

Data regarding the participants’ age and sex were extracted from their social identification numbers. Participants reported their parents’ educational levels, categorizing them as having completed primary education, secondary education, or college/university. Additionally, participants were queried about whether they had received financial aid from their parents. Of note, the items related to parents’ educational levels and parental financial support did not distinguish between biological and foster parents. Participants also reported if they had income-generating work during the past year (‘yes/no’). Financial difficulties were assessed by asking whether they had experienced difficulties affording basic living costs (such as for food, transportation and accommodation; ‘never’, ‘rarely’, ‘sometimes’, ‘often’) during the last 12 months, and participants were asked whether they had paid work during the last year (‘yes/no’). They were also asked about whether they had caregiving responsibilities for others (‘yes/no’).

#### 2.2.3. Academic Performance

Self-reported academic performance and incidences of failure were evaluated using three key metrics: (1) “Are you following your normed study progression (30 credits per semester) in the study program you are currently enrolled in?” with response options “no” or “yes”; (2) “Have you failed an exam since starting your studies at your college/university?” with responses categorized as “no,” “yes once,” or “yes, several times”; and (3) “What is your current GPA?” with grades ranging from A to fail (E/F). Participants reporting exam failures were further queried on the number of times they had failed.

#### 2.2.4. Mental and Somatic Health Problems

Symptoms of anxiety and depression were assessed using the Hopkins Symptoms Checklist (HSCL-25) [36], which is derived from the 90-item Symptom Checklist (SCL-90). The HSCL-25 serves as a screening tool designed to identify symptoms of anxiety and depression. It consists of a 10-item subscale for anxiety and a 15-item subscale for depression. Examples of items include “Nervous or restless”, “Have thoughts of taking your life”, and “Feeling hopeless for the future.” Each item is scored on a Likert scale ranging from 1 (not at all) to 4 (extremely) with the reference period covering the past month. The overall scale score is calculated by taking the mean of all 25 items, requiring all items to have valid scores to generate a mean score. Cronbach’s alpha for the HSCL-25 in this study was 0.94.

Somatic health was assessed using the Somatic Symptom Scale-8 (SSS-8) [37], which is an 8-item reliable and valid self-report measure originally derived from the well-validated Patient Health Questionnaire-15 (PHQ-15) [38]. The SSS-8 includes a general factor and four subscales, which cover gastrointestinal symptoms, pain, cardiopulmonary symptoms, and fatigue. Examples of items include “Chest pain or shortness of breath”, “Pain in arms, legs or joints” and “Feeling of tiredness or low energy”. Each item is scored on a Likert scale ranging from 0 (not at all) to 4 (very much) with the reference period covering the past week. The total score is calculated by summing all 8 items with a requirement for all items to have valid scores. The total scores on the SSS-8 range from 0 to 32. In this study, both the SSS-8 total score and factor scores were employed as continuous scores with higher scores indicating greater severity of somatic symptoms. Cronbach’s alpha for the SSS-8 in this study was 0.83.

#### 2.2.5. Loneliness

Loneliness was assessed using an abbreviated version of the widely used UCLA Loneliness Scale, which is known as “The Three-Item Loneliness Scale (T-ILS)” [39]. The T-ILS comprises three items, which are each rated on a 5-point Likert scale with the options “never,” “seldom,” “sometimes,” “often,” and “very often.” Participants were asked to indicate how often they experienced feelings of loneliness during the last year with the following prompts: (1) “How often do you feel that you lack companionship?” (2) “How often do you feel left out?” and (3) “How often do you feel isolated from others?” The T-ILS has demonstrated satisfactory reliability and possesses both concurrent and discriminant validity [39]. In this study, a continuous total score was calculated for the T-ILS, requiring all three items to have valid scores to generate a total score. Cronbach’s alpha for the T-ILS in this study was 0.87.

#### 2.2.6. Life Satisfaction

Life satisfaction was assessed using the Satisfaction With Life Scale (SWLS) [40]. The SWLS is a 5-item scale designed to measure global cognitive judgments of an individual’s satisfaction with their life rather than measuring positive or negative affective states. Participants responded to each item on a 7-point scale ranging from 1 (strongly disagree) to 7 (strongly agree), reflecting their level of agreement with each statement regarding their overall life satisfaction. Examples of items include “In most ways, my life is close to my ideal”, “My living conditions are very good”, and “So far, I have received the most important thing I have ever wished for in life”. In this study, a continuous total score was calculated for the SWLS, requiring all 5 items to have valid scores to generate a total score, ranging from 5 (low life satisfaction) to 35 (high life satisfaction). Cronbach’s alpha for the SWLS in this study was 0.89.

#### 2.2.7. Alcohol and Cannabis Use

The Alcohol Use Disorders Identification Test (AUDIT) is a widely used instrument developed by the World Health Organization to identify risky or harmful alcohol use [41,42]. Employed to detect potential alcohol-related problems, AUDIT comprises 10 items that assess various dimensions of alcohol consumption: the frequency, typical amount, and frequency of episodic heavy drinking (items 1–3); signs of alcohol dependence (items 4–6); and problems related to alcohol use (items 7–10). The AUDIT scores range from 0 to 40 with higher scores indicating greater severity of alcohol use issues. Example of items include “Do you drink 6 units of alcohol or more?”, “Have you felt guilty about drinking?”, and “Did you forget what happened the night before due to drinking?”.

Past-year cannabis use frequency was assessed with a single question: “How often did you use cannabis in the past 12 months?” with response options ranging from “never” to “daily”, including categories of “1 time”, “2–4 times”, “5–50 times”, “more than 50 times”, and “daily”. For analytical purposes, these categories were converted to T-scores to facilitate continuous analyses and ease comparisons across other continuous measures. Additionally, a dichotomous version was created where participants who reported using cannabis more than 50 times or daily were classified as at-risk users with those reporting less frequent use (1–50 times in the past year) serving as the reference category.

### 2.3. Ethics

The study was approved by the Regional Committee for Medical and Health Research Ethics in Western Norway (no. 2022/326437 [SHOT2022]). Written informed consent was obtained electronically after the participants had received a detailed introduction to the study.

### 2.4. Statistics

All statistical analyses were performed using SPSS (Version 29, IBM Corp, Armonk, NY, USA). Descriptive statistics were calculated to summarize the foster home and institutional characteristics within the study sample. Comparisons of sociodemographic and academic characteristics between students with foster care or institutional backgrounds and those without such experiences were conducted using chi-square tests for categorical variables and independent samples *t*-tests for continuous variables. Additionally, we examined group differences in mental and somatic health outcomes. To facilitate comparisons across instruments, sum scores were converted to standardized t-scores, and effect sizes between groups were calculated using Cohen’s d formula with pooled standard deviation. All tests were two-tailed, and a *p*-value of less than 0.05 was considered statistically significant. There was generally little missing data (<3%), and hence missing values were handled using listwise deletion.

## 3. Results

### 3.1. Placement Characteristics

As detailed in Table 1, a vast majority of the students (99.1%) had not experienced foster care or RYC. Among those who did, 382 students (0.64%) had lived solely in foster homes, while 84 students (0.14%) were solely in RYC, with a smaller proportion, 42 students (0.07%), experiencing both settings. Thus, the foster care alumni group comprises 376 (0.95%) respondents. The average number of foster home placements encountered by those in foster care was 1.5, suggesting a relatively stable placement scenario for most. Typically, the duration of stays in these foster homes was 6.7 years with the initial placement generally occurring around the age of 10 years. In contrast, RYC involved an average of 2.1 different placements per student with the average duration of stays being 2.6 years. The mean age at the first RYC placement was slightly higher at approximately 12.9 years. Gender differences were minimal but showed that males were slightly less likely than females to experience both foster homes and RYC settings.

### 3.2. Sociodemographic and Academic Characteristics

The sociodemographic and academic characteristics of the study sample, as detailed in Table 2, show some notable differences between students who have lived in foster homes or RYC and those who have not. Maternal and paternal education levels were significantly lower among foster care alumni students: only 39.4% of these students had mothers with a college or university education compared to 63.9% in the non-foster group. Similarly, 33.0% of students from foster or RYC care backgrounds had fathers with a college or university education in contrast to 54.9% among their non-foster peers. A larger proportion of the foster care alumni students reported having income-generating work last year (24.8% vs. 15.9%) and were less likely to receive financial help from their parents (21.3% vs. 41.9%). Moreover, they more frequently encountered financial problems, with 43% reporting they sometimes or often faced problems, compared to 24.3% of their peers. A significant proportion of students from foster care alumni had care responsibilities for others with 23.2% acknowledging care responsibilities, which was substantially higher than the 7.2% reported among non-foster peers.

Academic performance also differed; 38.1% of foster care alumni students achieved grades A or B, which was lower than the 49.6% observed in the non-foster group. The prevalence of failed exams was higher among foster care alumni students with 36.6% reporting one or more failures compared to 28.9% in the non-foster group. Delayed study progression was significantly more common among foster care alumni students at 13.6% versus 12.1% in their peers. Furthermore, a higher percentage of students from foster or RYC reported receiving special assistance or accommodations for their studies (11.4%) compared to those not from such backgrounds (6.7%). Notably, 25.0% of the foster care alumni students expressed an unmet need for special academic assistance, which was significantly higher than the 10.6% reported by their non-foster peers.

### 3.3. Mental and Somatic Health Problems

**Figure 1:**
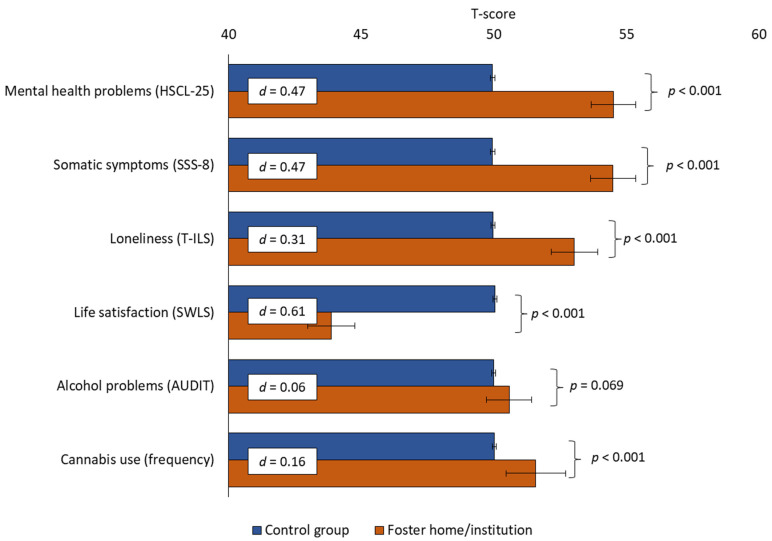
Mental and somatic health problems as well as substance use among foster care alumni students.

Figure 1 illustrates the mental and somatic health outcomes among foster care alumni students compared to the control group. Students with foster care backgrounds exhibited significantly higher levels of mental health problems and somatic symptoms each with a medium effect size of (d = 0.47, *p* < 0.001). Additionally, these students reported increased feelings of loneliness, with a small effect size of (d = 0.31, *p* < 0.001), and significantly lower life satisfaction scores with a medium effect size of (d = 0.61, *p* < 0.001). In contrast, differences in alcohol use were minimal and not statistically significant (d = 0.06, *p* = 0.069), highlighting a specific area where these groups do not differ markedly. However, the use of cannabis was significantly higher among the foster care alumni group (d = 0.16, *p* < 0.001). The proportion reporting using cannabis on a weekly to daily basis was 7.6% compared to 4.9% of their student peers, χ^2^ (1, *N* = 58,530) = 7.7, *p* = 0.005.

## 4. Discussion

Compared to their peers, foster care alumni students obtained somewhat lower grades and reported failing one or more exams more often. These differences were, however, marginal. No notable differences in study progression were observed. A substantially higher percentage reported receiving special assistance compared to their peers. Moreover, one quarter of the foster care alumni students reported unmet needs for assistance compared to one-tenth in the general sample. The foster care alumni group reported a substantially higher economic burden than others, which was characterized by a higher frequency of work income, more financial worries, and less financial support from parents. They also reported a higher load of informal care responsibilities, poorer mental and somatic health, more loneliness and lower life satisfaction. Notably, a higher proportion of foster care alumni students reported near daily cannabis use compared to their peers, indicating a pattern of more frequent and potentially risky behavior.

Overall, the foster care alumni group has a wide range of possible obstacles and risks related to poor academic functioning. Despite these challenges, in our study, this group still showed comparable study progression rates to peers. This is in contrast to a previous study that found slower progression rates among foster care alumni [8]. While our results are encouraging, we have not assessed graduation or drop-out rates which have been shown to be increased for foster care alumni [9,10], and future registry linkages of educational attainment are needed.

Notably, fewer of the foster care alumni in the current study reported having parents with a college/university degree compared to the general student sample. It is uncertain whether the foster care alumni group reports the educational status of their biological parents or their foster parents. Either way, our results are indicative of foster care alumni students as a group having a background where higher education is less of a norm than for students in general. This is somewhat expected, as foster parents tend to have lower educational attainment than the general adult population, suggesting that foster children are often raised in nonacademic families [15]. Moreover, among young adults with a history of foster care, their biological parents’ completion of upper secondary education is substantially lower than in the general population [43]. Also, parents with intellectual disability account for 20–25% of all custody cases [44]. Taken together, this indicates that for some young adults with a history of out-of-home care, the parental educational disadvantages may have been present from an early age. Our findings imply that the group of foster care alumni enrolled in higher education is not a group singled out by a particularly high representation of parental higher education.

One of our striking findings is that across several indicators, foster care alumni students reported a higher economic burden than that of their peers. Most notably, the proportion receiving economic aid from parents was only 21%, which was half of that in the general student sample. This aligns with our finding of a substantially higher percentage in this group who reported work income relative to the general sample. The developmental phase of emerging adulthood is more often than not characterized by ongoing dependence of parental support: relationally, practically and financially [45]. Direct parental support, i.e., monetary transfers, is indeed associated with higher occupational status for college graduates [46]. For young adults transitioning out of foster care, this phase may be characterized by an abrupt halt to relational and practical support. The vulnerability of this period for care leavers have gained attention internationally, and a variety of policies to extend support for this group has been implemented in several countries [47]. The outcome of extended care policies shows mixed results, however [48,49,50]. Although predicting enrollment into college, there seems to be no effect of extended foster care on degree of college completion [51,52]. In Norway, after-care is mandatory to offer all youths receiving support from the CWS before the age of 18 up until the age of 25 [53]. Financial support is commonly used as after-care, especially up until completion of upper secondary school. Approximately two out of three of youth in out-of-home care receive after care after the age of 18, but by the age of 21 years, the proportion has reduced by over 50% [54]. One may argue that the Norwegian policy of tuition-free admission increases access to higher education for economically underprivileged groups. Still, our results show that foster care alumni students experience considerable worries about their economic situation. This could be due to the experienced lack of safety net that many of their student peers consider their parents to be. Our results point to the need for a stronger policy to aid foster care alumni in their pursuit of higher education in terms of enduring financial support to ameliorate the financial disadvantages due to lack of parental figures with capacity to support emerging adults with a history of out-of-home care.

The foster care alumni group report substantially higher somatic and mental health problems and lower quality of life compared to their student peers. Across high-income countries, the prevalence of mental disorders among children and youth in the child welfare system is strikingly similar with a point prevalence of 50% [21]. A recent meta-analysis concludes that prevalence rates persist for adults with an out-of-home placement history, and were significantly higher than in the general population [31]. Hence, our findings are somewhat expected. One could, however, argue that the proportion of foster care alumni who enter higher education was expected to present with good health relative to other adults with an out-of-home placement history. Our results indicate that this might not be the case. The finding of high somatic and mental health problems in this group despite adequate academic performance nuances the concept of resilience as a unidimensional outcome for this group.

The heightened score on somatic symptoms (gastrointestinal symptoms, pain, cardiopulmonary symptoms, and fatigue) relative to the general student population may be seen in the context of the heightened exposure to adverse childhood experiences in this group. Adults with a history of exposure to trauma are twice as likely to have a functional somatic syndrome [55]. Moreover, exposure to childhood maltreatment increases the risk of somatic central sensitivity syndromes such as fibromyalgia, chronic fatigue syndrome, chronic lower back pain, restless leg syndrome and irritable bowel syndrome in young adulthood [56]. There are strong arguments for viewing child maltreatment as a root cause of both mental and pain-related symptoms. These experiences occur during the developmental phase of the maturing brain, and they may offset dysfunctional threat detection, fear processing, and reward/anti-reward mechanisms, yielding physical and mental health problems in adulthood [57]. It is worth noticing that our study indicates that the high burden of mental and somatic health symptoms is present also among those foster care alumni who shows substantial resilience in that they have succeeded in entering higher education.

Notably, more foster care alumni students reported having responsibilities as young carers compared with their student peers. In the general student population, informal care responsibilities are associated with a negative impact on study progress and the number of failed exams [32]. This aligns with our finding of a slightly higher frequency of failed exams among foster care alumni; however, our group does not stand out regarding delayed study progression compared to their student peers. In general, students with informal care responsibilities report more mental health problems, insomnia, somatic symptoms, and lower life satisfaction [58]. Moreover, young adult carers are more likely to feel lonely [32]. The foster care alumni students seem to stand out both in terms of more often being a young adult carer and reporting more loneliness than their peers. Whether the respondent feels isolated from others is one of the probes of loneliness in this study. Being a young adult with experiences of alternate care, the adverse life experiences associated with this, and the ongoing role as a young adult carer may contribute to elevated scores of loneliness. In sum, these life experiences may be difficult to share with peers and may lead to the feeling of isolation. These findings of increased feelings of loneliness are important also for future outcomes for this group. Among foster care alumni graduating from college, the during-college life domains that have been deemed important for post-college outcomes were supportive relationships and community connections together with physical and mental health [34]. In this context, our findings of a comparably large group reporting daily intake of cannabis among foster care alumni students are worrisome, pointing to a subgroup of foster care alumni students that may lack sufficiently adaptive coping strategies to deal with the obstacles and strain evidently present in this group. These students are at particular risk of impaired academic performance [59,60].

Of note, almost twice as many foster care alumni students report receiving special assistance/accommodations in their studies as their student peers. Moreover, 25% report unmet needs for assistance compared to 11% in the general student sample. Taken together, this is a strong indicator that students with a background from out-of-home care experience high needs of academic support and that these needs are often unmet. This finding may be seen in the context of lower parental education, less financial support, and higher informal care responsibilities among foster care alumni students. Further, they have a higher rate of mental and somatic health problems that could qualify them for special assistance. Taken together, our findings leave the impression that compared to their peers, the student life of foster care alumni comes with high demands of practical/economic independence and abilities to manage care responsibilities, which is often in combination with having less of an academic background as a foundation for their own pursuit of higher education. In this regard, it is not surprising that a relatively large percentage report unmet needs for support. To meet the needs of this group, further studies are needed to assess in depth which components such assistance should entail.

### Strengths and Limitations

One strength of this study is the large, national sample of students in the survey, including the large sample of foster care alumni students. To our knowledge, this is the first European study to investigate how foster care alumni fare as students compared to their peers with use of a wide range of standardized measures of health and well-being and academic outcomes.

However, some limitations should be noted. A national sample yields generalizability within the context of Norwegian students. But higher education is tuition-free in Norway, and the organization and mandate of the child welfare services varies greatly between countries as well as within Europe. This may decrease the generalizability of our findings to countries outside Scandinavia. Another limitation concerns the representativeness of the sample. Despite the large sample of nearly 60,000 students, the overall response rate was moderate at 35.1%. This is somewhat low for web-based surveys among students in this age group [61]. Furthermore, we do not know the response rate among our target group. 

In the questions on parental education and parental economic support, we did not differentiate between foster parents and parents of origin. Thus, we do not know for certain whom these foster care alumni students were referring to when answering. Nevertheless, the impact of parental education is predominantly a social factor, and therefore, the answers here may be valid either way, since the participants are probably referring to the carers most prominent in their representation of “parent”.

Additionally, the imbalance in sample sizes between foster care alumni and non-foster home groups may impact the statistical power and interpretation of our findings, potentially affecting the robustness of comparisons made between these groups.

Finally, the current study is cross-sectional, and future studies should follow these students into graduation or drop-out and ideally into working life. Although we included a wide range of factors that may impact educational outcomes, the relatively large sample size still provides limited statistical power to explore how specific factors relate to particular academic outcomes.

## 5. Conclusions

Using a national survey targeting all students in higher education in Norway, our study has shed light on how foster care alumni students fare compared to their peers. By employing standardized, validated measures, we have identified a broad range of factors relevant to outcomes in higher education. Overall, the foster care alumni student group faces a wide range of possible obstacles and risks related to poor academic functioning. Compared to their peers, they experience poorer health and well-being, a higher burden of care, and substantially more economic strain and worries. Altogether, this paints a picture of everyday life characterized by undue hardship for students with a background in out-of-home care.

Despite these challenges, their academic performance remains remarkably resilient. This suggests a high level of resourcefulness with foster care alumni managing significant strain while still maintaining study progression. Our findings contribute substantial knowledge on the resources available and the pitfalls present for the completion of higher education for young adults leaving care. To establish the linkage of these risk factors to outcomes, future registry linkages of educational attainment for foster care alumni students are needed.

Early prevention efforts aimed at improving mental and somatic health among youth in foster care, as well as financial and social support, are crucial to achieving the goal of promoting higher education attainment for all youth raised in out-of-home care.

## Figures and Tables

**Table 1:** Placement characteristics of the study sample.

	Women	Men	Total
None, *n* (%)	39,201 (99.0%)	19,835 (99.3%)	59,036 (99.1%)
Residential youth care (RYC) only, *n* (%)	65 (0.16%)	19 (0.10%)	84 (0.14%)
Foster home only, *n* (%)	280 (0.71%)	102 (0.51%)	382 (0.64%)
Foster home and RYC, *n* (%)	31 (0.08%)	11 (0.06%)	42 (0.07%)
^#^ foster homes, mean (SD)	1.5 (0.7)	1.5 (0.8)	1.5 (0.7)
1 foster home, *n* (%)	192 (65.3%)	75 (70.1%)	267 (66.6%)
2 foster homes, *n* (%)	79 (26.9%)	20 (18.7%)	99 (24.7%)
3 foster homes, *n* (%)	12 (5.1%)	8 (7.5%)	23 (5.7%)
3 foster homes, *n* (%)	8 (2.7%)	4 (3.7%)	12 (3.0%)
Foster home stay duration ^#^, mean (SD)	6.6 (5.32)	7.0 (5.5)	6.7 (5.4)
Age when moved to foster home, mean (SD)	10.1 (5.5)	9.6 (5.2)	10.0 (5.4)
^#^ RYC, mean (SD)	2.1 (1.2)	2.0 (1.1)	2.1 (1.2)
1 foster home, *n* (%)	42 (44.7%)	13 (44.8%)	55 (44.7%)
2 foster homes, *n* (%)	23 (24.5%)	7 (24.1%)	30 (24.4%)
3 foster homes, *n* (%)	7 (7.4%)	5 (17.2%)	12 (9.8%)
3 foster homes, *n* (%)	22 (23.4%)	4 (13.8%)	26 (21.1%)
RYC stay duration ^#^, mean (SD)	2.6 (2.8)	2.8 (2.2)	2.6 (2.7)
Age when moved to RYC, mean (SD)	13.3 (3.6)	11.8 (4.7)	12.9 (4.0)

# Length of stay in years (single or longest).

**Table 2:** Sociodemographic and academic characteristics of the study sample.

	No Foster Home/Institution	Foster Home/Institution	*p*-Value *	Total
Females, % (*n*)	66.4% (39,201)	74.0% (376)	<0.001	66.5% (39,577)
Age, mean (SD)	26.1 (7.3)	27.7 (7.7)	<0.001	26.1 (7.3)
Maternal education, % (*n*)				
Primary	6.2% (3518)	24.4% (97)		6.3% (3615)
Secondary	30.0% (17,064)	37.2% (154)		30.0% (17,218)
College/university	63.9% (36,370)	39.4% (163)		63.7% (36,533)
Paternal education, % (*n*)			<0.001	
Primary	7.5% (4184)	23.1% (84)		7.6% (4268)
Secondary	37.6% (20,985)	44.0% (160)		37.6% (21,145)
College/university	54.9% (30,680)	33.0% (120)		54.8% (30,800)
Income-generating work last year (yes), % (*n*)	15.9% (9327)	24.8% (126)	<0.001	15.9% (9453)
Financial help from parents (yes), % (*n*)	41.9% (24,506)	21.3% (108)	<0.001	41.7% (24,614)
Financial problems, % (*n*)			<0.001	
Never	55.1% (32,335)	33.4% (169)		54.9% (32,504)
Rarely	20.6% (12,091)	23.5% (119)		20.6% (12,210)
Sometimes	18.4% (10,810)	29.6% (150)		18.5% (10,960)
Often	5.9% (3460)	13.4% (68)		6.0% (3528)
Care of others (yes), % (*n*)	7.2% (4234)	23.2% (118)	<0.001	7.3% (4352)
Academic performance (GPA), % (*n*)			<0.001	
A	9.6% (5082)	6.1% (28)		9.5% (5110)
B	40.0% (21,253)	32.0% (147)		39.9% (21,400)
C	38.7% (20,572)	43.7% (201)		38.8% (20,773)
D	9.8% (5213)	14.3% (66)		9.9% (5279)
E/F	1.9% (1012)	3.9% (18)		1.9% (1030)
Failed exams, % (*n*)			<0.001	
No	71.1% (41,774)	63.4% (321)		71.0% (42,095)
Yes, once	14.9% (8761)	18.6% (94)		14.9% (8855)
Yes, several times	14.0% (8207)	18.0% (91)		14.0% (8098)
Delayed study progression (yes), % (*n*)	12.1% (3569)	13.6% (69)	<0.001	12.1% (7172)
Do you receive special assistance/ accommodations in your studies? % (*n*)				
Yes	6.7% (3920)	11.4% (58)	<0.001	6.7% (3978)
No, but I need it	10.6% (6235)	25.0% (127)		10.8% (6362)

* *p*-values were calculated using chi-squared tests.

## Data Availability

Norwegian data protection regulations and GDPR impose restrictions on sharing of individual participant data. However, researchers may gain access to survey participant data by contacting the publication committee (borge.sivertsen@fhi.no). Approval from the Norwegian Regional Committee for Medical and Health Research Ethics (https://rekportalen.no/) is a pre-requirement for access to the data. The dataset is administrated by the NIPH, and guidelines for access to data are found at https://www.fhi.no/en/more/access-to-data.

## References

[1] Salmi J., D’Addio A. (2021). Policies for achieving inclusion in higher education. Policy Rev. High. Educ..

[2] Tupan-Wenno M., Camilleri A.F., Fröhlich M., King S. (2016). Effective Approaches to Enhancing The Social Dimension of Higher Education.

[3] Dworsky A., Pérez A. (2010). Helping former foster youth graduate from college through campus support programs. Child. Youth Serv. Rev..

[4] Vinnerljung B., Hjern A. (2011). Cognitive, educational and self-support outcomes of long-term foster care versus adoption. A Swedish national cohort study. Child. Youth Serv. Rev..

[5] Statistics Norway Statistics Norway. Status for Elevar 22 år Eller Yngre Etter Avslutta Grunnskole, Etter Antall år Etter Avslutta Grunnskole, Status, Kjønn, Barnevernstiltak. https://www.ssb.no/statbank/table/13349/tableViewLayout1/.

[6] Greger H.K., Stuifbergen M.C., Jozefiak T., Kayed N.S., Lydersen S., Rimehaug T., Schalinski I., Seim A.R., Singstad M.T., Wallander J. (2024). Young Adults with a History of Residential Youth Care: A Cohort Profile of a Hard-to-Reach Population. Int. J. Environ. Res. Public Health.

[7] Backe-Hansen E., Madsen C., Kristofersen L.B., Hvinden B. (2014). Barnevern i Norge 1990–2010. En Longitudinell Studie.

[8] Gillum N.L., Lindsay T., Murray F.L., Wells P. (2016). A Review of Research on College Educational Outcomes of Students Who Experienced Foster Care. J. Public Child Welf..

[9] Pecora P.J., Williams J., Kessler R.C., Hiripi E., O’Brien K., Emerson J., Herrick M.A., Torres D. (2006). Assessing the educational achievements of adults who were formerly placed in family foster care. Child Fam. Soc. Work..

[10] White C.R., O’brien K., Pecora P.J., Buher A. (2015). Mental Health and Educational Outcomes for Youth Transitioning from Foster Care in Michigan. Fam. Soc..

[11] Font S., Palmer L. (2024). Left behind? Educational disadvantage, child protection, and foster care. Child Abus. Negl..

[12] Day A., Dworsky A., Fogarty K., Damashek A. (2011). An examination of post-secondary retention and graduation among foster care youth enrolled in a four-year university. Child. Youth Serv. Rev..

[13] Unrau Y.A., Font S.A., Rawls G. (2012). Readiness for college engagement among students who have aged out of foster care. Child. Youth Serv. Rev..

[14] Askeland K.G., Radlick R.L., BØe T., Hysing M., La Greca A.M., Nilsen S.A. (2024). Parental unemployment and educational outcomes in late adolescence: The importance of family cohesion, parental education, and family income in a Norwegian study. Scand. J. Public Health.

[15] Berlin M., Vinnerljung B., Hjern A., Brännström L. (2019). Educational outcomes of children from long-term foster care: Does foster parents’ educational attainment matter?. Dev. Child Welf..

[16] Okpych N.J., Courtney M.E. (2021). Barriers to Degree Completion for College Students With Foster Care Histories: Results From a 10-Year Longitudinal Study. J. Coll. Stud. Retent. Res. Theory Pract..

[17] Batsche C., Hart S., Ort R., Armstrong M., Strozier A., Hummer V. (2014). Post-secondary transitions of youth emancipated from foster care. Child Fam. Soc. Work..

[18] Hass M., Allen Q., Amoah M. (2014). Turning points and resilience of academically successful foster youth. Child. Youth Serv. Rev..

[19] Johnson R.M. (2021). The state of research on undergraduate youth formerly in foster care: A systematic review of the literature. J. Divers. High. Educ..

[20] Lehmann S., Havik O., Havik T., Heiervang E. (2013). Mental disorders in foster children: A study of prevalence, comorbidity and risk factors. Child Adolesc. Psychiatry Ment. Health.

[21] Bronsard G., Alessandrini M., Fond G., Loundou A., Auquier P., Tordjman S., Boyer L. (2016). The Prevalence of Mental Disorders Among Children and Adolescents in the Child Welfare System: A Systematic Review and Meta-Analysis. Medicine.

[22] Jozefiak T., Kayed N.S., Rimehaug T., Wormdal A.K., Brubakk A.M., Wichstrøm L. (2016). Prevalence and comorbidity of mental disorders among adolescents living in residential youth care. Eur. Child Adolesc. Psychiatry.

[23] Vinnerljung B., Hjern A. (2018). Health Care in Europe for Children in Societal Out-of-Home Care.

[24] Larsen M., Goemans A., Baste V., Wilderjans T.F., Lehmann S. (2021). Predictors of quality of life among youths in foster care—A 5-year prospective follow-up study. Qual. Life Res..

[25] Greger H.K., Myhre A.K., Lydersen S., Jozefiak T. (2016). Child maltreatment and quality of life: A study of adolescents in residential care. Health Qual. Life Outcomes.

[26] Hjorth C.F., Bilgrav L., Frandsen L.S., Overgaard C., Torp-Pedersen C., Nielsen B., Bøggild H. (2016). Mental health and school dropout across educational levels and genders: A 4.8-year follow-up study. BMC Public Health.

[27] Bengtsson T.T., Olsen R.F., Lausten M. (2022). The role of mental health problems in out-of-home care youths’ educational pathways: Quantitative and qualitative analysis of Danish longitudinal data. Child Abus. Negl..

[28] Salazar A.M., Jones K.R., Emerson J.C., Mucha L. (2016). Postsecondary strengths, challenges, and supports experienced by foster care alumni college graduates. J. Coll. Stud. Dev..

[29] Watt T.T., Norton C.L., Jones C. (2013). Designing a campus support program for foster care alumni: Preliminary evidence for a strengths framework. Child. Youth Serv. Rev..

[30] Kind N., Seker S., d’Huart D., Bürgin D., Jenkel N., Boonmann C., Habersaat S., Urben S., Fegert J.M., Clemens V. (2023). High-risk substance use and psychosocial functioning in young adult care leavers: Findings from a 10-year follow-up study. Child. Youth Serv. Rev..

[31] Seker S., Boonmann C., Gerger H., Jäggi L., d’Huart D., Schmeck K., Schmid M. (2022). Mental disorders among adults formerly in out-of-home care: A systematic review and meta-analysis of longitudinal studies. Eur. Child Adolesc. Psychiatry.

[32] Haugland B.S.M., Hysing M., Sivertsen B. (2022). Study progress, recreational activities, and loneliness in young adult carers: A national student survey. BMC Psychol..

[33] Hysing M., Petrie K.J., Bøe T., Lønning K.J., Sivertsen B. (2020). Only the Lonely: A Study of Loneliness Among University Students in Norway. Clin. Psychol. Eur..

[34] Salazar A.M., Schelbe L. (2021). Factors associated with post-college success for foster care alumni college graduates. Child. Youth Serv. Rev..

[35] Sivertsen B., Råkil H., Munkvik E., Lønning K.J. (2019). Cohort profile: The SHoT-study, a national health and well-being survey of Norwegian university students. BMJ Open.

[36] Derogatis L.R., Lipman R.S., Rickels K., Uhlenhuth E.H., Covi L. (1974). The Hopkins Symptom Checklist (HSCL): A self-report symptom inventory. Behav. Sci..

[37] Gierk B., Kohlmann S., Kroenke K., Spangenberg L., Zenger M., Brahler E., Lowe B. (2014). The somatic symptom scale-8 (SSS-8): A brief measure of somatic symptom burden. JAMA Intern. Med..

[38] Kroenke K., Spitzer R.L., Williams J.B.W. (2002). The PHQ-15: Validity of a New Measure for Evaluating the Severity of Somatic Symptoms. Psychosom. Med..

[39] Hughes M.E., Waite L.J., Hawkley L.C., Cacioppo J.T. (2004). A Short Scale for Measuring Loneliness in Large Surveys: Results From Two Population-Based Studies. Res. Aging.

[40] Diener E., Emmons R.A., Larsen R.J., Griffin S. (1985). The Satisfaction With Life Scale. J. Personal. Assess..

[41] Saunders J.B., Aasland O.G., Babor T.F., De la Fuente J.R., Grant M. (1993). Development of the Alcohol Use Disorders Identification Test (AUDIT): WHO Collaborative Project on Early Detection of Persons with Harmful Alcohol Consumption-II. Addiction.

[42] Babor T.F., Higgins-Biddle J.C., Saunders J.B., Monteiro M.G. (2001). AUDIT: The Alcohol Use Disorders Identification Test: Guidelines for Use in Primary Health Care.

[43] Paulsen V., Thoresen S.H., Wendelborg C. (2023). Outcomes in adulthood among former child welfare services recipients: Findings from a Norwegian registry study covering two decades. Eur. J. Soc. Work..

[44] Tøssebro J., Midjo T., Paulsen V., Berg B. (2017). Prevalence, Trends and Custody Among Children of Parents with Intellectual Disabilities in Norway. J. Appl. Res. Intellect. Disabil..

[45] Johnson M.K., Benson J. (2011). The implications of family context for the transition to adulthood. Early Adulthood in a Family Context.

[46] Manzoni A. (2018). Parental Support and Youth Occupational Attainment: Help or Hindrance?. J. Youth Adolesc..

[47] van Breda A.D., Munro E.R., Gilligan R., Anghel R., Harder A., Incarnato M., Mann-Feder V., Refaeli T., Stohler R., Storø J. (2020). Extended care: Global dialogue on policy, practice and research. Child. Youth Serv. Rev..

[48] Courtney M.E. (2019). The benefits of extending state care to young adults. Leaving Care and The Transition to Adulthood: International Contributions to Theory, Research, and Practice.

[49] Doucet M.M., Greeson J.K.P., Eldeeb N. (2022). Independent living programs and services for youth ‘aging out’ of care in Canada and the U.S.: A systematic review. Child. Youth Serv. Rev..

[50] Gunawardena N., Stich C. (2021). Interventions for young people aging out of the child welfare system: A systematic literature review. Child. Youth Serv. Rev..

[51] Courtney M.E., Okpych N.J., Park S. (2018). Report from CalYOUTH: Findings on the Relationship Between Extended Foster Care and Youth’s Outcomes at Age 21.

[52] Okpych N.J., Courtney M.E. (2020). The relationship between extended foster care and college outcomes for foster care alumni. J. Public Child Welfar.

[53] (2021). Lov Om Barnevern. https://lovdata.no/dokument/NL/lov/2021-06-18-97.

[54] Paulsen V., Wendelborg C., Riise A., Berg B., Tøssebro J., Caspersen J. (2020). Ettervern-en God Overgang Til Voksenlivet?: Helhetlig Oppfølging Av Ungdom Med Barnevernerfaring.

[55] Afari N., Ahumada S.M., Wright L.J., Mostoufi S., Golnari G., Reis V., Cuneo J.G. (2014). Psychological Trauma and Functional Somatic Syndromes: A Systematic Review and Meta-Analysis. Psychosom. Med..

[56] Chandan J.S., Keerthy D., Zemedikun D.T., Okoth K., Gokhale K.M., Raza K., Bandyopadhyay S., Taylor J., Nirantharakumar K. (2020). The association between exposure to childhood maltreatment and the subsequent development of functional somatic and visceral pain syndromes. Eclinicalmedicine.

[57] Cay M., Gonzalez-Heydrich J., Teicher M.H., van der Heijden H., Ongur D., Shinn A.K., Upadhyay J. (2022). Childhood maltreatment and its role in the development of pain and psychopathology. Lancet Child Adolesc. Health.

[58] Haugland B.S.M., Hysing M., Sivertsen B. (2020). The Burden of Care: A National Survey on the Prevalence, Demographic Characteristics and Health Problems Among Young Adult Carers Attending Higher Education in Norway. Front. Psychol..

[59] Meda S.A., Gueorguieva R.V., Pittman B., Rosen R.R., Aslanzadeh F., Tennen H., Leen S., Hawkins K., Raskin S., Wood R.M. (2017). Longitudinal influence of alcohol and marijuana use on academic performance in college students. PLoS ONE.

[60] Suerken C.K., Reboussin B.A., Egan K.L., Sutfin E.L., Wagoner K.G., Spangler J., Wolfson M. (2016). Marijuana use trajectories and academic outcomes among college students. Drug Alcohol Depend..

[61] Wu M.-J., Zhao K., Fils-Aime F. (2022). Response rates of online surveys in published research: A meta-analysis. Comput. Hum. Behav. Rep..

